# Gardening—The Great Test of Health

**Published:** 1986-06

**Authors:** T. R. Steen

**Affiliations:** Emeritus Consultant Anaesthetist, Southmead Hospital, Bristol


### 'Gardening—the Great Test of Health.'

**by T. R. Steen MB. BChir, Camb. FFARCS.** Emeritus Consultant Anaesthetist, Southmead Hospital, Bristol

*'When I look into the Fishponds in my Garden,*
*Methinks I see a Thing, arm'd with a Rake,*
*That seems to strike at me'.*
*– The Duchess of Malfi. (Act 5, scene 5, line 5).*

How justly has John Webster been called the most perceptive of the Jacobean dramatists! The Eternal Verity quoted above is familiar to every victim of the Common (or Garden) Rake injury. The implement under advisement is lying, spikes upward, and you tread on it, Whereupon, in literally striking demonstration of the principles of leverage and dynamics, the handle springs up, with noteworthy acceleration and force, in an elegant arc directed precisely at your head. Only if actual concussion and retrograde amnesia ensue, can the experience fail to be unforgettably painful.

Elementary, you will rightly say. Indeed, since Webster's time, the armamentarium and impedimenta of horticulture have reached a terrifying degree of sophistication, and one trembles to think what he might tell us now. Consider, if you dare, the simple Flymo, so craftily designed to amputate the hallux, or the potentialities for electrocution in your workaday hedge-trimmer, not to mention those noxious weed-killers which insidiously impair sanity itself (vid inf). Californian millionaires, we learn, not rarely meet their end when paralysed by a stroke while riding the motor-mower: inexorably, the machine carries them on into the swimming-pool, to drown. Nasty enough, though maybe not as bad as drowning in liquid manure, which has also been described.

Week after week, Obituary Notices in the BMJ attest the poet's cry:–

'You are nearer Death's Door in a garden
than anywhere else on God's Earth', for gardening is by far the most frequently mentioned hobby of the deceased. Presumably on the grounds that such propaganda speaks for itself, the BMA has not launched, as in the case of smoking, a virulent vendetta against this hazardous habit. Nevertheless, for the general public who may not regularly see the BMJ, a Health Warning on every gardening appurtenance would surely not come amiss.

In Bristol we are favoured by the memory of our Most Famous Citizen, the late Dr W. G. Grace, a man of proven physique if ever there was one, yet tragically found dead in his garden, still only 66. And which of us has not heard of the daring horticultural researches undertaken by, significantly, our distinguished Professor of Pathology himself? To some at least, the message has come through, and I think particularly of a friend living out on the fringe of wildest Gloucestershire, equipped only with his native courage, his flame-thrower and his cement-mixer, 'pushing back' (in Housman's telling phrase) 'the frontiers of darkness', an inspiration to us all.

My ignorance of Botany, of which I make no secret, may derive from imperfect teaching at school. In preparation for a display in the Science Department, my master instructed me to try out Hortomone A, a fertilizer much advertised at the time. One box of seedlings was treated with this nostrum, while another was not. As D-Day approached, I was told, 'For a clearer effect, pick the small plants out of this box and the big ones out of the other'. Though gratified by the praise of the Headmaster who, duly impressed, resolved to purchase the stuff forthwith, even at that tender age I thought the whole thing fishy. And my pathetic attempt to extend my knowledge in the holidays by asking the park-keeper, 'Excuse me, does that flower belong to the Primula Family?', met with the stern rebuff, 'That flower belongs to the London County Council'. Moreover, when it came to the actual practice of gardening, from early youth I found it so stressful and alarming that, long before the Feminists began to tell us such things, I formulated the unshakeable belief that no man should have a garden bigger than his wife can conveniently handle.

Thus rigorously prepared, I was ready, at the time of the last General Election but about five, for my Moment of Truth. Like so many Great Discoveries (the case of Fleming and Penicillin leaps to mind), this one depended on an almost trivial observation by someone exceptionally qualified to discern its significance. In response to some Socialist cracks about a 'candyfloss economy', the Conservative Central Office saw fit to announce that there were 13 million gardeners in Britain. At the same time the cost of the NHS topped £1,000 million per annum. Younger readers may not appreciate how revolutionary, at the time, was the question I then dared to pose. Could it be that these two facts were more than co-incidental? Could all this gardening, – so far from being, as glibly implied, wholesome evidence of widespread vigorous health – actually be itself a major, possibly even *the* major, cause of disease?

Till then, in my pristine innocence, as an anaesthetist inquiring into the general health of patients facing surgery, I had always interpreted as favourable the common reply, 'Still get the odd bit of gardening done, doctor', croaked with a brave smile from the poor, ravaged old body. How poignant to realise that, unknowingly, the patient was almost certainly identifying just what had brought him to his present pass!

At about the same time, the Urologists discovered Trabeculation. Whereas I used to wonder what the cystoscopists were up to and when they might stop, it was now no longer a case of 'Captain, art thou sleeping there below?', for racy chatter from the nether regions kept me well-informed of the state of play. And I very soon noted an odd fact:– Trabeculation is never seen in sea-faring folk! I became so sure of this that, while an orchid-grower unsurprisingly exhibited trabeculation of such severity as actually to reach the supra-pubic skin, I was temporarily baffled when a man, giving his occupation as professional gardener, proved to have absolutely no trabeculation at all! However, subsequent careful inquiry revealed that, during the Great War, he had served in the Navy, which must have saved him from this fell complaint. What an object-lesson in the importance of detailed and accurate history-taking!

Now this was the corollary to clinch my hypothesis. For, if gardening causes disease, then non-gardening must prevent it. And where, on the surface of the globe, can one be more free from gardening than on board ship? (I refer to ocean-going vessels only: inland waterways are, to coin a phrase, another kettle of fish, for one

* In one such case the author assisted the editor in a laparatomy for an acute abdominal emergency. Ed.

has seen canal-boats with window-boxes). It is to this that we must attribute the proverbial good health of Jolly Jack Tar.

To pursue the nautical motif, it is small wonder that, with our great maritime tradition, the hymn 'For Those at Sea' enjoys a revered place in our national life. But, while almost unbearably moving when rendered at, say, Plymouth, it may not carry the same impact at Sutton Coldfield or Ashby-de-la-Zouche. For, numerous though our mariners are, our gardeners are more numerous still, and there ought to be a hymn for them too, with some such haunting refrain as:–

'Protecting whereso'er they go
All those in hazard with the hoe'.

On this pious note, Biblical scholars will recall the case of Adam who, permanently excluded from gardening at an early age, lived to be 130 (Genesis. V,3).

Hardly any aspect of life escapes a horticultural component. Thus, devotees of music know that Handel's Largo comes from an aria sung in his garden by the King of Persia, hoping that his plants may be free from blight and other contingencies: it is so sad that, not surprisingly, the original production ran for only five performances, though the mournful strains have since fittingly won lasting acceptability at funerals. Musicians know too that the year 1863 saw a momentous change in our social history, when 'Home, Sweet Home', which had been Top of the Pops for 37 years, was displaced by 'Come into the Garden, Maud', thus initiating the gardening craze which has afflicted us ever since, together with that turbulent dichotomy of House v Garden culminating in the song 'The Garden's full of furniture and the House is full of plants': (which latter phenomenon causes the wary visitor to many a modern home to reach for his malaria tablets). The decade following 1863, be it noted, saw the description, for the first time, of some very horrendous illnesses, including Amyotrophic Lateral Sclerosis and Posterior Inferior Cerebellar Thrombosis.

Visitors to Bath will notice a particularly fine garden-shop: bang opposite, they have built a whole Hospital for Rheumatic Diseases! Less exotically, near even the humblest garden-shop you will always find a Chemist's: if ever you don't, why not start a Chemist's there and make your fortune? A shrewd ploy would be to market Gardener's First Aid Outfits of increasing complexity, like the old Meccano sets, beginning with a Kiddie's Kit of a couple of bandages and some back-ache liniment, and working up to the Postgraduate Set, complete with stomach pump, Thomas's splints, iron-lung and defibrillator.

Very gingerly, having noted that his profession was recorded as 'gardener', I asked a patient, 'How do you keep in your general health?' Imagine my alarm when he immediately sat bolt upright, fixed me with a piercing glare and yelled, 'My mind's been poisoned!' 'Whatever do you mean?', I managed to gasp. 'Weed-killers!', he screamed, 'I was going off my head. Luckily there was a clever doctor who knew what it was'.

Now for something from the other end of the spectrum. 'I hate gardening so much, I've even crazy-paved my window-box', said a robust young man in reply to the well-trained houseman's tactful inquiry about his habits. This 'patient' had, it transpired, got into hospital by mistake. In some case-notes by the same houseman, even I, with all my experience, was startled to read. 'Evidence of gardening in the Right Iliac Fossa': only later did I discern that the operative word was 'guarding'.

This brings us to the problem of Classification, so beloved of purists. To them one must say that Classification of Diseases is easy enough: there are *a.* diseases due to gardening and *b.* other diseases. As to the classification of gardening itself, I recommend the system traditionally applied to obstetric haemorrhage:– Primary/Secondary, True/False, Concealed/Revealed. To take an extreme example, if someone in the prime of life were to go into his garden and, coram populo, actually do some gardening, that would be Primary, True and Revealed. Conversely, if an elderly person, trying to 'keep up' with the neighbours, were to pretend to grow mushrooms in a disused underground air-raid shelter, that would be Secondary, False and Concealed.

To conclude this brief review of a vast subject, I can only distribute, as it were, a few random forkfuls from the rich compost of my experience:–

*a.* *Torsion of the Gall-Bladder*, not a pretty sight, is mercifully rare. It afflicts old ladies of 86 after dragging the hose across the lawn.*

*b.* Primulas have already been mentioned. They are a potent cause of *Eczema*, as was discovered by the whole family of a Consultant who received a gift of these flora from his well-meaning Registrar. It is a measure of this Consultant's generosity of spirit that he readily supported the early promotion of that self-same Registrar to a senior post, elsewhere.

*c.* In a recent article, allegedly by a Dermatologist, my eye fell on the astonishing words, 'We do not know the cause of *Vitiligo*'. How ignorant can you get, for goodness sake? Though not a dermatologist, I learned way back in my student days that the cause of Vitiligo is Privet Hedges, and I soon had ample confirmation. An Operating-Theatre Attendant had, in his garden, a privet hedge so immense that he needed a step-ladder to cut it. Eventually he fell in and had to be rescued by the Fire Brigade. There then developed such dreadful vitiligo that he had to transfer to an office-job and, within 25 years, he was dead. Only 83.

To addicts who assert that they have gardened for years without ill-effect, one must concede that, if true, this certainly proves the soundness of their original constitution. At the same time, real friends must be candid and will not fail to remind them of the astute dictum of George Eliot:–

'The length of time during which a given event has not happened, though often cited as proof that it will never happen, may in fact be the very condition which makes that event imminent', for Gardening (—like Pregnancy, as I was taught in my very first lesson on Ante-Natal Care) is and always will be a Great Test of Health!

STOP PRESS (14th April). Prince Charles arrived in Vienna with his finger bandaged and arm in a sling, following his unfortunate injury, while planting a tree in his garden about 3 weeks ago.

